# The Impact of the Consistency Evaluation Policy of Generic Drugs on R&D Investment Intensity of Pharmaceutical Companies—An Empirical Study Based on the Difference-in-Differences Model

**DOI:** 10.3389/fpubh.2022.902025

**Published:** 2022-06-09

**Authors:** Yanyi Wei, Jialin Zhu, Jiahui Qiao, Dawei Zhang, Yuwen Chen

**Affiliations:** ^1^School of Business Administration, Shenyang Pharmaceutical University, Shenyang, China; ^2^Liaoning Province Pharmaceutical and Health Industry Development Strategy Research Base, Shenyang, China

**Keywords:** the consistent evaluation policy of generic drugs, R&D investment intensity, placebo test, operational nature, regional distribution, profitability

## Abstract

In 2016, China began to execute the consistency evaluation policy of generic drugs. Many scholars believed that the policy would stimulate pharmaceutical firms to increase R&D investment with a theoretical perspective, but few have conducted empirical studies. Therefore, we conduct a difference-in-differences (DID) model and use panel data of 111 A-share listed pharmaceutical firms from 2012 to 2020 to empirically study the impact of the consistency evaluation policy of generic drugs on pharmaceutical firms' R&D investment intensity. The result shows that the policy has a significant positive impact on the R&D investment intensity of firms with chemical generics, robust under the test for parallel trend test, placebo test, and the propensity score matching and difference-in-differences (PSM-DID) test. In addition, we further analyzed the impact of this policy on the R&D intensity of pharmaceutical firms according to the heterogeneity of enterprise's operational nature, regional distribution and profitability. From the perspective of time changes and the average effect, the R&D investment intensity of private pharmaceutical firms is more affected by the policy than state-owned enterprises; the R&D investment intensity of pharmaceutical firms in the eastern region is more affected by this policy than those in the central and the western; the R&D investment intensity of high-profitability pharmaceutical firms is more affected by the policy than those with low-profitability. The consistency evaluation policy is still being implemented, and its impact on pharmaceutical firms needs to be studied from different empirical research perspectives in the future.

## Introduction

Since the release of the “National Drug Safety 12th Five-Year Plan” in 2012 (1), the development goal of China's pharmaceutical manufacturing industry has changed from “pursuing quantity” to “improving quality.” And then, the government has successively issued a series of policies to improve the quality of medicines. Among them, the “Generic Drug Consistency Evaluation Policy” is the policy that has the most profound impact on the pharmaceutical manufacturing industry. In March 2016, the State Council issued the Opinions on Carrying out the Quality and Efficacy Consistency Evaluation of Generic Drugs (referred to as the “Opinions”) (2), asking the approved generic drugs to be re-evaluated in stages and batches by the principle of consistency with the quality and efficacy of the original drugs. The Generic consistency evaluation is a history missing lesson make-up in the generic drug supervision in China, and it is also a reference from the supervision of the drugs in Japan and the United States (3).

The starting point of the Chinese government's implementation of the generic drug consistency evaluation policy is to improve the quality of generic drugs. During the implementation of the policy, some companies with low-quality generic drugs will be eliminated. Hence, this policy became a vital tool for the government to reshuffle the industry and lead the transformation and upgrading of the pharmaceutical industry. Due to considerable technical barriers in imitation of some original drugs, those pharmaceutical firms that have long relied on low-end replication may give up participating in the consistency evaluation of generic drugs and face elimination. In contrast, some pharmaceutical firms will actively participate in the evaluation, strengthen the research and development of high-end generic drugs, and use the generic drug consistency evaluation policy to increase their drug market share further. Moreover, some other pharmaceutical firms will be forced to transform and research innovative drugs. In this way, the consistent evaluation policy of generic drugs will change the pattern of China's pharmaceutical market. Some Chinese scholars have theoretically proposed that the generic drug consistency evaluation policy can promote pharmaceutical enterprises to increase R&D investment (4, 5). Therefore, we intend to use an empirical analysis method to verify this view to examine the real impact of the generic drug consistency evaluation policy on the R&D investment of pharmaceutical firms.

We take 111 A-share listed pharmaceutical firms as the research object and use the Difference-in-Differences method to conduct an empirical study on this impact. The study found that the generic drug consistency evaluation policy has a significant role in promoting the R&D investment intensity of pharmaceutical firms. Possible contributions of this paper include: This paper uses an empirical analysis method to test the impact of the generic drug consistency evaluation policy on the R&D investment of enterprises and enrich the research on the factors affecting enterprise innovation. This article enriches the evaluation of generic drug consistency evaluation policies from an empirical research perspective. This paper examines the differences in the operational nature, regional distribution, and profitability of the impact on the firm's R&D investment by the generic drug consistency evaluation policy. It enriches the research on the difference in the effect of the generic drug consistency evaluation policy.

The remainder of this study is divided into the following areas: “Institutional Background” provides a systematic review of the generic drug consistency evaluation policy; “Research Hypothesis” presents our main research questions; “Materials and Methods” describes Sample Selection and Data, Variables, and Equation Design; “Results” presents Descriptive Statistic, Baseline Empirical Results and Robustness Checks. The “Conclusion and Discussion” section summarizes and discusses the above content.

## Institutional Background

The Opinions released in March 2016 opened the prelude to the consistent evaluation of generic drugs. Since then, the generic drugs marketed before implementing the new registration classification of chemical drugs, those that have not been reviewed according to the principle of the quality and efficacy consistent with the original drugs, must undergo consistency evaluation, including domestic generic drugs, imported generic drugs, and localized varieties of original research drugs. The evaluation method is mainly based on bioequivalence experiments. When bioequivalence is met, the generic drug is considered “equivalent” to the original brand drug (6), so the quality of generic drugs that have passed the evaluation is higher than that have not. The strategy adopted in the consistency evaluation of generic drugs in China is to implement in stages and continuously expand the range of varieties. The evaluation of oral-solid preparations is carried out first, followed by the review of injections, and finally, the assessment of other dosage forms (7).

In the first stage, the solid chemical preparations were mainly evaluated. And the first evaluation batch was the oral-solid preparations of generic chemical drugs approved in the *National Essential Medicines List of China (2012 edition)* before October 1, 2007, which should be completed by the end of 2018. The oral-solid preparations in the *List* that have particular circumstances and need to carry out clinical effectiveness experiments should be completed the evaluation by the end of 2021.

Due to the difficulty in selecting reference preparations and the tight evaluation time, most pharmaceutical companies initially waited and watched the development of consistency evaluation and did not participate in the evaluation. In order to ensure the smooth progress of the consistency evaluation, the relevant ministries and commissions have upgraded and revised the *National Essential Medicines List* (8), prioritizing the inclusion of varieties that have passed the consistency evaluation into the *List* while removing the varieties that have not passed. After implementing the new version of the *List (2018 edition)* on November 1, 2018, there will no longer be a unified evaluation time limit requirement for the varieties included in *List* (9). In addition, the government has been issuing a series of supporting measures (see the Supplementary Material) to help pharmaceutical enterprises solve corresponding difficulties.

Under the vital impetus of the government, the consistent evaluation of generic drugs entered the second stage in 2020, that is, the variety range of generics in the evaluation has been extended to chemical injections (10). The chemical injection generics that have not been approved according to the principle that the quality and efficacy consistent with original drugs need to be re-evaluated. Although there is no longer a unified evaluation time limit requirement, when the same generic drug from many pharmaceutical companies participated in the evaluation, other enterprises needed to complete the evaluation within 3 years after the first enterprise passed the consistency evaluation. Otherwise, the generic drug will not be allowed to be re-registered.

**Figure 1:**
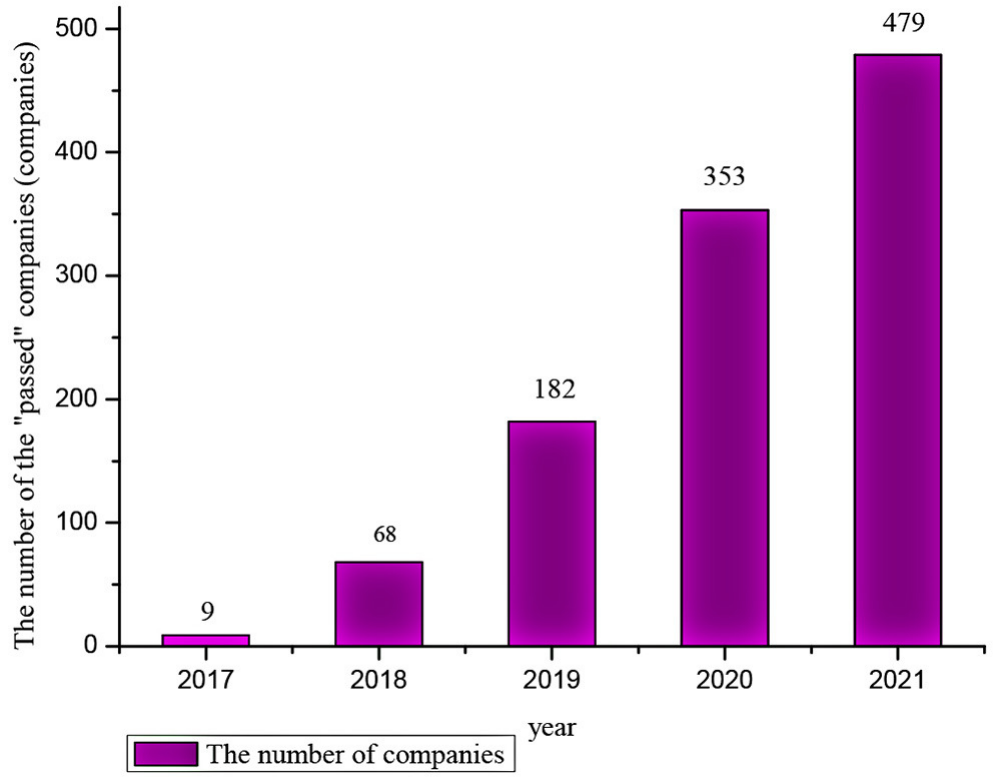
The number of companies that pass the consistency evaluation each year (including “deemed to pass”). “Deemed to pass”: In addition to the generic drugs that have passed the consistency evaluation, it also includes generic drugs marketed in Europe, America, and Japan, and generic drugs marketed according to the original registration classification that meets the requirements of the consistency evaluation (11) (Data from “Chinese List of Chemical Drugs,” https://www.cde.org.cn).

With the advancement of the policy, the number of chemical -generic drugs that have passed the evaluation has increased. As of December 31, 2021, the number of generic drugs that have passed the consistent evaluation has reached 1,822 (data from https://www.cde.org.cn), involving more than 400 varieties. The number of solid preparations and injections that have passed the evaluation is about 3:1. At the same time, the number of enterprises through generic drug consistency evaluation increased year by year (as shown in Figure 1), including some foreign pharmaceutical companies in China that passed the consistency evaluation. The evaluation of generic drugs will enter a new stage in the future, the range of varieties will be expanded to other dosage forms, and more pharmaceutical will participate in the evaluation.

## Research Hypothesis

Some scholars believe that the implementation of the generic drug consistency evaluation policy can not only improve the quality of drugs but also improve the level of R&D and production technology in the pharmaceutical industry and promote the transformation of China's pharmaceutical industry from imitation to innovation (12, 13). On the one hand, the government has successively issued a series of supporting policies, including the incentive policies of relevant institutions in many provinces in China to subsidize the firms that have passed the evaluation with funds. For example, Anhui Province rewards RMB 1 million for a single generic drug product that has passed the evaluation; Sichuan Province improves the medical insurance payment standard, giving a subsidy of up to RMB 2 million (14). More and more pharmaceutical enterprises have participated in this work, the competition among domestic pharmaceutical companies has become more intense, and the companies would face more tremendous financial pressure (13). On the other hand, the benchmark condition for generic drugs to participate in centralized procurement is that they have passed the consistency evaluation. Generally, no more than three enterprises win the bid for the same drug (15, 16). If pharmaceutical firms do not pass the consistency evaluation, their market position will decline, and they will lose the original market share. In theory, in order to pass the evaluation and stabilize their market position or expand the market share, many pharmaceutical firms will increase R&D investment. Some strong firms will invest more money in developing high-end generic drugs to occupy the market share of high-end generics. The imitation technology barriers of high-end generic drugs are high, with a monopoly advantage. Some other firms have withdrawn from the fierce competition in generic drugs consistency evaluation and increased R&D investment to develop original research drugs, even though the cost of originator drugs development strategy is higher than the generic drugs evaluation strategy. Under this policy, more and more firms realize that R&D investment is crucial in promoting firms' economic growth and development (17). Innovation-driven firm development has become the choice of more firms.

This is the independent choice of pharmaceutical companies under government intervention from a macro perspective. Government intervention is also one of the critical factors affecting innovation (18). Government intervention, such as government subsidy policies and preferential tax policies, is often used to promote China's pharmaceutical industry's innovation and development (19, 20). The generic drug consistency evaluation policy is also a government intervention that can promote enterprise innovation. The consistency evaluation of generic drugs is a kind of directive intervention to guide the development direction of enterprises. In this context, the pharmaceutical market competition is more intense, and pharmaceutical companies understand that only by choosing to improve the quality of drugs can they become winners. Therefore, whether the pharmaceutical firms participate in the consistency evaluation or not, they need to increase R&D investment and innovate continuously to ensure the long-term development of enterprises in the fierce competition.

In addition, from the perspective of heterogeneity analysis, the R&D investment of China's pharmaceutical companies would vary due to the different operation nature, geographical differences, or different profitability. Wang (21) conducted an empirical study on the innovation behavior of different holding companies and believed that state-owned enterprises have more advantages in innovation investment than various holding companies. Similarly, through empirical research, Shi et al. (22) believed that the comprehensive technical efficiency of innovation activities of state-owned pharmaceutical enterprises is higher than that of private enterprises. Wang et al. (23) used the DEA model to compare the technological innovation of the pharmaceutical manufacturing industry in the eastern, central, and western regions of China and found that the technological innovation efficiency of the pharmaceutical manufacturing industry in the eastern and western regions was higher than that in the central region. Wang et al. (24) used the DEA-RAM model to study the unified efficiency of innovation's green performance in 29 sub-sectors of China's manufacturing industry. They found an apparent disparity in the unified efficiency between China's western and eastern regions. Chen and Lee (25) used the VAR model to analyze the dynamic relationship among cash flow, profitability, and R&D investment of 588 Chinese listed companies. The results showed that the higher the profitability of the enterprise, the higher the R&D intensity. Dalvadi and Mansuri (26) studied relevant data of 16 Indian pharmaceutical companies from June 2005 to 2014 and concluded that profitability significantly affects R&D spending.

Therefore, this paper puts forward the following hypothesis:

H1: The generic drugs consistency evaluation policy can encourage generic pharmaceutical firms to increase R&D investment;

H2: The impact of the generic drug consistency evaluation policy on the R&D investment intensity of pharmaceutical companies is different in state-owned and private enterprises.

H3: The impact of the generic drug consistency evaluation policy on the R&D investment intensity of pharmaceutical companies is different in the eastern, central and western regions.

H4: The impact of the generic drug consistency evaluation policy on the R&D investment intensity of pharmaceutical companies may be more significant for companies with high profitability.

## Materials and Methods

### Sample Selection and Data

Based on the latest individual stock classification released by Shenyin & Wanguo (http://www.swsresearch.com/, which is always provides authoritative industry classification standards) in July 2021, we selected the three sub-sectors belongs to the pharmaceutical and biological plate—chemical-pharmaceutical firms, traditional Chinese medicine firms, and biopharmaceutical firms as the research object. The main businesses of pharmaceutical companies in the other three sub-sectors are pharmaceutical commerce, medical devices, and medical services. Because the generic drug consistency evaluation policy mainly impacts pharmaceutical manufacturers, these sub-sectors are not studied. The selected firms in these three sub-sectors are all A-share pharmaceutical listed firms. Most firms in the pharmaceutical and biological sector did not disclose their R&D investment before 2012, and we collected the data samples from 2012 to the end of 2020. The data samples we collected are all from China Stock Market & Accounting Research Database (CSMAR, http://cndata1.csmar.com/) and the annual reports of pharmaceutical listed firms. Furthermore, we selected data samples for empirical analysis based on the following principles: (1) Excluding samples of firms marked with ST or ^*^ST (stocks subject to delisting risk warning); (2) Excluding samples of firms with missing data on essential indicators in annual reports and incomplete disclosure of relevant data; (3) Excluding samples of firms with abnormal data; (4) Excluding samples of firms listed after December 31, 2012; (5) In order to reduce the influence of abnormal extreme values, this paper carries out 1 and 99% tail reduction processing for the continuous variables involved. Then, we selected 111 A-share pharmaceutical listed firms for research, we conducted an empirical analysis based on the financial-related data of these firms from 2012 to 2020, a total of 999 observations.

### Variables

#### Dependent Variable (RDI)

The core explained variable of this paper is R&D investment intensity, it is represented by the ratio of the listed company's R&D investment to the its operating income in that year, reflecting the actual R&D investment situation of firms of different sizes (27).

#### Independent Variable (Treat_it_ × Post_it_)

We used the consistent evaluation policy of generic drugs as the core explanatory variable, it is a dummy variable. The full name of this policy is “The Quality and efficacy consistency evaluation of generic drugs” or “The re-evaluation of quality and efficacy of registered generic medicines.” According to the policy, the main task is to evaluate the quality and efficacy of chemical-generic drugs, the chemical-generics mainly include solid preparations and chemical injections, and some domestic traditional Chinese medicine companies or biopharmaceutical companies also produce chemical-generics. Therefore, we borrowed the practice of Si (28) and selected pharmaceutical companies that produce chemical-generic drugs as the treatment group in this study.

The selection of the treatment and control group in this paper is based on the 111 A-share listed pharmaceutical companies. There is no official channel to disclose the list of the pharmaceutical companies that participated in the consistency evaluation now, so we focused on the Chinese List of Chemical Drugs (https://www.cde.org.cn), and we looked for the list of “Medicines that have passed the Generic Drug Consistency Evaluation,” to compare the companies in the list with 111 companies selected by us, and then we took pharmaceutical companies with matching names such as Hengrui, Fosun, Huahai and other pharmaceutical companies as the treatment group; In addition, considering that companies not listed but producing chemical-generic drugs will also be affected by the policy, we retrieved the 2016–2020 annual financial reports of the remaining companies from the website *http://www.cninfo.com.cn*, we put the companies whose operation product categories and objectives involve chemical-generic drugs as the treatment group. For example, the Chinese medicine firm Guizhou YIBAI and the biopharmaceutical firm Staidson clearly stated in their annual reports that they would strengthen the research and development of high-end chemical-generic drugs. We got 66 companies in the treatment group, including 45 chemical-pharmaceutical firms, 18 traditional Chinese medicine firms, and three biopharmaceutical firms. The remaining 45 firms served as the control group, including ten chemical-pharmaceutical firms, 26 traditional Chinese medicine firms, and nine biopharmaceutical firms. These firms, such as Zhejiang NHU company, produce the chemical bulk drug, Dong-E-E-Jiao produce traditional Chinese medicines, and “Changchun High-tech” produce biological drugs or biosimilars. Their products do not involve chemical generics, and there is currently no pressure from generic consistency evaluation.

Then we set the dummy variable of the treatment group as *Treat*_*it*_. When the firm belongs to the treatment group, it is affected by the policy, *Treat*_*it*_ equals 1, and when the firm belongs to the control group, *Treat*_*it*_ equals 0. And we used the time dummy variable *Post*_*it*_ to represent the occurrence of the policy. In 2016, the generic drug consistency evaluation policy was implemented, so *Post*_*it*_ equals 1 after 2016; otherwise, *Post*_*it*_ equals 0. The core explanatory variable is related to both the treatment group dummy variable (*Treat*_*it*_) and the time dummy variable (*Post*_*it*_), so it is set to *Treat*_*it*_ × *Post*_*it*_, which is used to measure the net impact of the policy.

#### Control Variables (X_it_)

Based on existing research (29–32), we selected seven factors that may affect the R&D investment of pharmaceutical firms as control variables, among them, the enterprise market competitiveness is represented by the industry Lerner index. Rojas (33) once stated that the Lerner index is an important indicator representing competitiveness because it specifies the monopoly position of an enterprise in the market. The Lerner index closer to 1, the stronger the enterprise's competitiveness in the market will be. After implementing the consistency evaluation policy, the competition of domestic pharmaceutical enterprises will become more intense, which would affect the companies' R&D intensity. Therefore, we added this variable in our study.

The detailed description of all variables is shown in Table 1.

**Table 1:** Research variables.

**Variable type**	**Variable name**	**Description**	**Variable symbol**
Dependent variable	R&D investment intensity	R&D investment/operation income	*RDI*
Independent variable	The consistent evaluation policy	Policy treated virtual variables × Time virtual variables	*Treat_*it*_ × Post_*it*_*
Control variables (*X_*it*_*)	Enterprise size	ln (Total assets)	*Size*
	Capital structure	Debt/Total assets	*Lev*
	Cash ratio	Cash from operating activities/Total asset	*Cash*
	Return on equity	Net profit/Stockholders' equity balance	*Roe*
	Company's growth ability	Operating income growth rate	*Growth*
	Ownership concentration	Proportion of the largest shareholder	*Shareh1*
	Company's market competitiveness	Industry Lerner index	*Comp*

### Equation Design

We constructed a double difference model to test the impact of the policy on the R&D investment intensity of pharmaceutical firms by the dummy variable. The equation is as follows:

(1) $$\begin{array}{l} RDI_{it}=\alpha +\beta^{*}Treat_{it}\times Post_{it}+\theta X_{it}+\lambda_{i}+\upsilon_{t}+\varepsilon_{it} \end{array}$$

$$\begin{array}{l} Treat_{it}=\{\begin{array}{l} 0,\;company\;i\;belongs\;to\;the\;firm\;in\;the\;control\;group\\ 1,company\;i\;belongs\;to\;the\;firm\;in\;the\;treatment\;group\; \end{array} \end{array}$$

$$\begin{array}{r} Post_{it}=\{\begin{array}{c} 0,\;t<2016\;(The\;policy\;not\;implemented)\\ 1,\;t\geq 2016\;(The\;policy\;implemented) \end{array} \end{array}$$

This DID model is one of the more mature empirical methods in the field of policy evaluation, it avoids endogenous problems to a large extent, and it helps us study the net effect of the policy. In equation (1) the coefficient β measures the net effect of the consistency evaluation policy of generic drugs on R&D investment, *X*_*it*_ represents a set of control variables composed of other internal factors that may affect R&D investment, λ_*i*_ represents the individual fixed effect of the company, υ_*t*_ represents the year fixed effect, ε_*it*_ is a random disturbance term.

## Results

### Descriptive Statistic

Table 2 records the descriptive statistical results of the variables in our study. On the level of panel data, the amount of R&D investment intensity *(RDI*) varies significantly among different A-share listed pharmaceutical firms. The lowest *RDI* is 0.36%, and the highest *RDI* is 17.99%. Judging from the mean and standard deviation (SD), the differences in Size of different pharmaceutical companies are minor. However, the SDs for Capital structure (*Lev*), Company growth ability (*Growth*), and Ownership concentration (*Shareh1*) show that they change significantly over time. The empirical analysis uses the natural logarithm of Size and the percentages of the rest variables.

**Table 2:** Descriptive statistics results.

**Variable**	***N***	**Mean**	**SD**	**Min**	**Max**
*RDI*	999	4.545	3.225	0.360	17.990
*Size*	999	22.224	0.933	20.283	24.502
*Lev*	999	32.890	18.253	3.945	82.091
*Cash*	999	6.352	5.728	−9.883	21.593
*Roe*	999	8.772	8.901	−32.153	34.313
*Growth*	999	13.765	24.477	−42.522	126.793
*Shareh1*	999	33.348	13.409	9.560	69.160
*Comp*	999	15.144	3.416	2.584	19.376

### Baseline Empirical Results

This section mainly assesses the actual impact of implementing the quality and efficacy consistency evaluation policy of generic drugs on the R&D investment intensity of pharmaceutical firms with chemical generics. To control potential heteroskedasticity and autocorrelation issues, we use robust standard errors of province level clustering (34). According to the model established in this paper, we regress the sample data with and without control variables, respectively, and obtain the corresponding results in columns (1) and (2) of Table 3. Colum (1) reveal the immediate results without control variables. The estimated coefficient of the cross term *Treat*_*it*_ × *Post*_*it*_ is 1.209, which is significant at the statistical level of 1 %.; the Colum (2) shows the result adding control variables, the estimated coefficient of *Treat*_*it*_ × *Post*_*it*_ is still positive statistically significant at the level of 1%. It means that the policy has a positively affect the R&D investment intensity of pharmaceutical firms with chemical generics. Hypothesis H1 is supported, that is, the consistency evaluation policy of generic drugs can effectively stimulate firms to increase R&D investment and effectively induce enterprises to increase R&D innovation.

**Table 3:** Benchmark regression results.

	**(1)**	**(2)**
*Treat_*it*_ × Post_*it*_*	1.209^***^	1.117^***^
	(5.35)	(5.06)
		(2.60)
*Constant*	4.145^***^	−4.752
	(55.50)	(−0.44)
Observations	999	999
R-squared	0.750	0.776
Control variables	No	Yes
Company FE	Yes	Yes
Year FE	Yes	Yes

*Robust t-statistics in parentheses,*

*** *p < 0.01*.

### Robustness Checks

The credibility of the baseline regression results of the difference-in-difference model needs a series of validity tests, and we mainly test model (1): parallel trend test, placebo test, and PSM-DID test, through which we confirm that the baseline regression results are robust.

#### Parallel Trend Test

In general, using the DID model to measure the net effect of the policy requires testing the premise hypothesis. The premise of DID model in this paper is that the changing trend of R&D investment intensity was the same in the treatment and control groups before the consistency evaluation policy of generic drugs was released. Therefore, we draw on the research methods of Liu and Qiu (35) and Lyu et al. (36) to further test the changing trends of the treatment and control groups, and the empirical model set as follows:

(2) $$\begin{array}{r} RDI_{it}=\alpha +\beta_{k}\sum_{k\geq -4}^{4+}\;Treat{,}_{i}\times Post{,}_{2016+k}+\theta X_{it}+\lambda_{i}\\ +\upsilon_{t}+\varepsilon_{it} \end{array}$$

**Figure 2:**
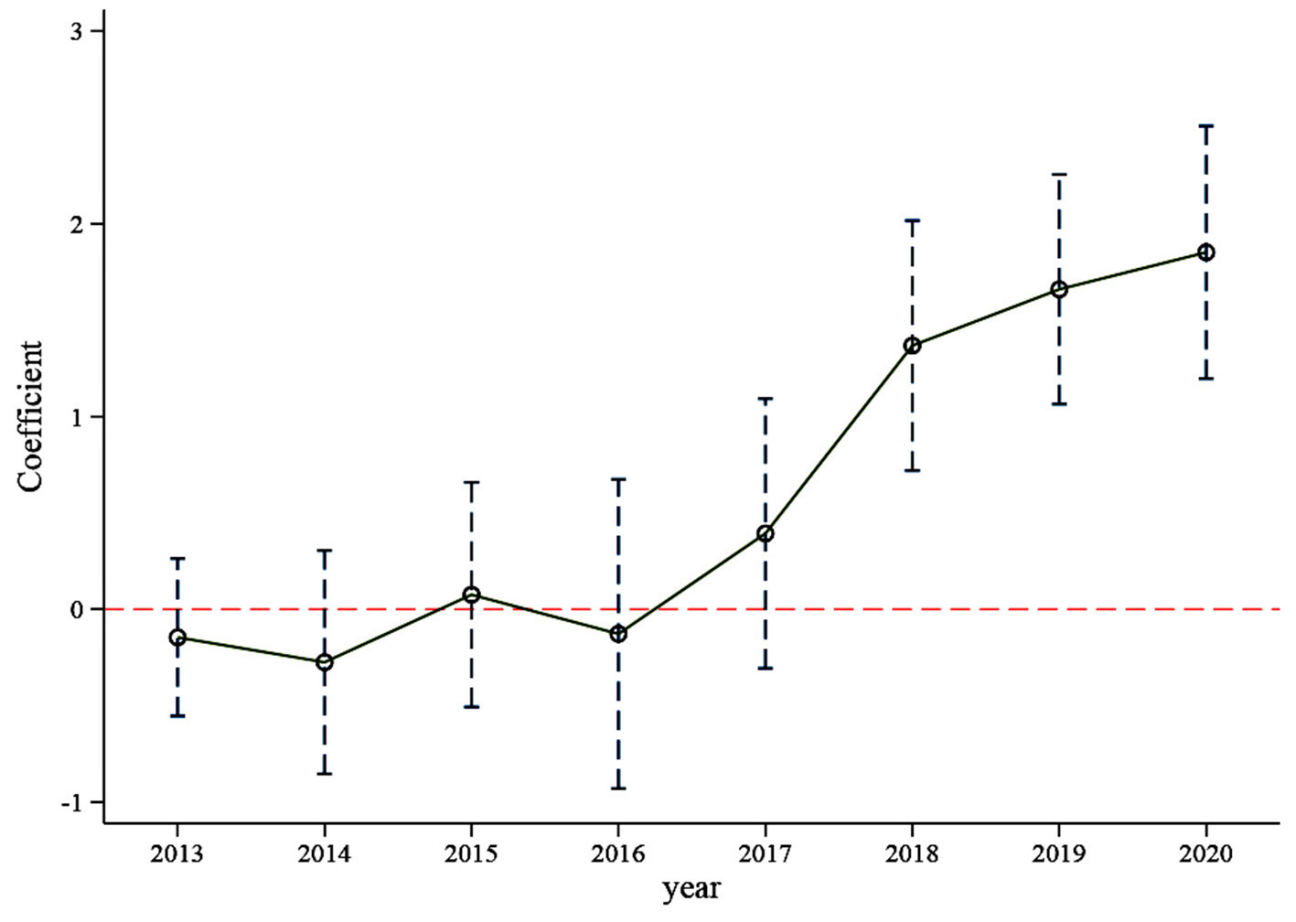
Parallel trend test (with control variables).

We tested the changing trend of R&D investment intensity in the 3 years before and5 years after implementing the policy. This paper used the year 2012 as the benchmark period and the result of the base period will be deleted. As the Figure 2 shows, the regression coefficient of each year was near the 0 axes before implementing the policy in 2016 and not significant. This means that there was no significant difference in the trend of R&D investment intensity between the treatment and control groups before implementing the policy, indicating that the samples selected in this paper passed the parallel trend test.

At the same time, it can be seen from Table 4, regardless of whether control variables are added or not, the regression coefficient of the dummy variable of 2016 is not significant; without the control variables, the dummy variable of 2017 is significant, but after adding the control variables, the coefficient of the dummy variable of 2017 is not significant. That means adding control variables can reduce the effect of endogeneity. After 2018, the coefficient values of the dummy variable in each year increase and are all significant (*p* < 0.01), indicating that the policy had a lagging impact on R&D investment, this result is consistent with reality. The policy was launched in 2016, and some pharmaceutical firms began to prepare to participate in the consistent evaluation of generic drugs. Because the firms had no experience selecting reference preparations and conducting bioequivalence experiments, the consistency evaluation work was not smooth at the early stage. Moreover, many pharmaceutical firms are still in a wait-and-see state. The initial evaluation time limit eliminates the enthusiasm of some firms, but with the improvement of a series of supporting policies and the cancellation of the unified evaluation time limit, more and more firms participate in the consistent evaluation of generic drugs. The generic drug market is becoming more and more fierce, and pharmaceutical firms continue to increase R&D investment. We can infer from Figure 2 that the generic drug consistency evaluation policy will have a significant and continuous positive impact on the intensity of R&D investment.

**Table 4:** Parallel trend test results.

	**(1)**	**(2)**
*Treat_, *i*_ × Post,_2013_*	−0.045	−0.146
	(−0.22)	(−0.61)
*Treat_, *i*_ × Post,_2014_*	−0.133	−0.275
	(−0.41)	(−0.81)
*Treat_, *i*_ × Post,_2015_*	0.060	0.075
	(0.19)	(0.22)
*Treat_, *i*_ × Post,_2016_*	0.110	−0.128
	(0.28)	(−0.27)
*Treat_, *i*_ × Post,_2017_*	0.610^*^	0.393
	(1.85)	(0.96)
*Treat_, *i*_ × Post,_2018_*	1.416^***^	1.368^***^
	(3.85)	(3.59)
*Treat_, *i*_ × Post,_2019_*	1.717^***^	1.660^***^
	(4.45)	(4.74)
*Treat_, *i*_ × Post,_2020_*	2.045^***^	1.852^***^
	(4.54)	(4.81)
*Constant*	4.163^***^	−6.213
	(36.46)	(-0.58)
Observations	999	999
R-squared	0.757	0.783
Control variables	No	Yes
Company FE	Yes	Yes
Year FE	Yes	Yes

*Robust t-statistics in parentheses,*

*** *p < 0.01,*

* *p < 0.1*.

#### Placebo Test

**Figure 3:**
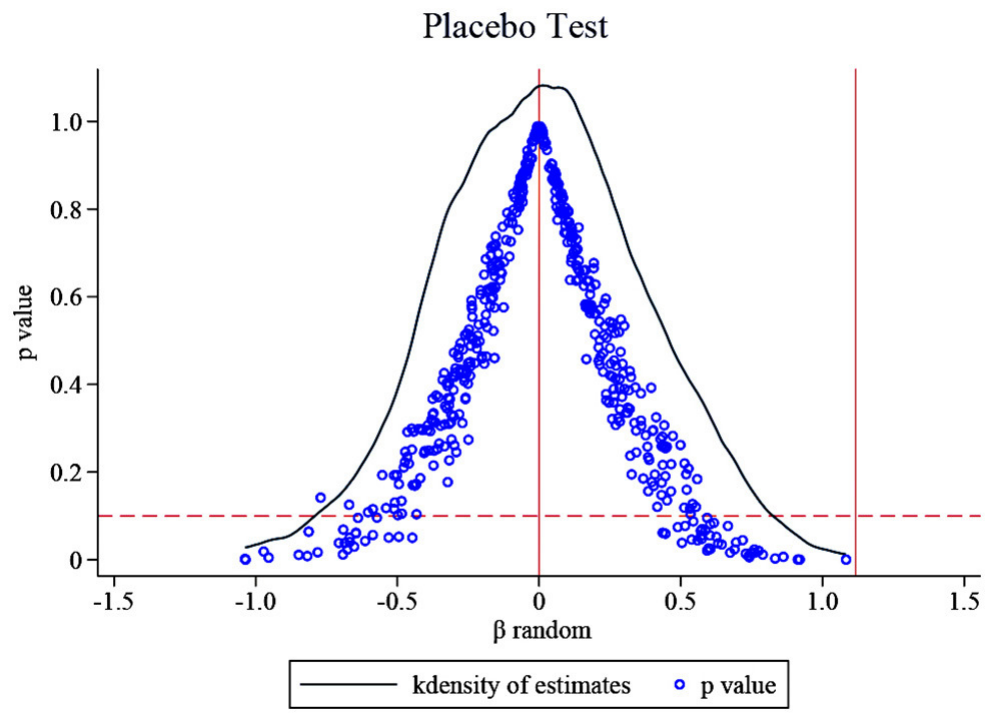
The results of the placebo test.

Testing for the influence of unobservable factors. The benchmark regression results in this paper are obtained under the fixed effects of the company and the year. To a certain extent, the factors that change the firm over time are controlled, but there may still be other characteristics of the firm that are difficult to observe. Therefore, this part draws on the idea of La Ferrara et al. (37), Liu and Lu (38) and Cai et al. (39) to indirectly test whether the non-direct observable characteristics will affect the benchmark regression results. We keep the policy shock time as 2016 and test the baseline empirical results of this paper by randomly selecting treatment groups. The research sample in this paper contains 111 companies, of which 66 are treatment groups. Therefore, we randomly selected 66 listed pharmaceutical companies from 111 companies as the “fake” treatment group, and the remaining 45 companies were set as the control group to construct a dummy variable ${Treat}_{it}^{fake}$ for the placebo test, then we construct the cross term ${Treat}_{it}^{fake}$ **×** ***Post***_***it***_ for placebo test. Since the “fake” treatment group is randomly generated, the cross term used for the placebo test will not significantly impact the R&D investment intensity of listed pharmaceutical companies, so the coefficient of ${Treat}_{it}^{fake}$ **×** ***Post***_***it***_ should be 0 (i.e., ***β***^random^ **= 0**). That is to say, if not affected by other unobservable factors, the regression coefficient of ${Treat}_{it}^{fake}$ **×** ***Post***_***it***_ will not significantly deviate from zero; otherwise, it proves that the benchmark regression results are wrong. This paper conducts 500 random samplings to avoid the interference of small probability events, correspondingly, 500 ***β***^random^ were generated, and the results were recorded in Figure 3. The figure also shows the distribution of 500 corresponding P values, and the rightmost vertical line the true estimated coefficient 1.117. It can be seen that the ***β***^random^ of the 500 random processes are concentrated around zero, most *P* > 0.1, that is, most ***β***^random^ are not significant. The mean value of ***β***^random^ is 0.009, which is very close to zero and not significant. Moreover, the actual regression result's estimated coefficient is 1.117, significantly different from the coefficient value in placebo inspection. These prove that non-observed factors will not affect the benchmark regression results. The benchmark regression results are robust. The increase in R&D investment of chemical generic drug firms is indeed the role of the generic drug consistency evaluation policy.

#### PSM-DID Test

Considering that there may be factors outside the policy that will have an impact on R&D investment, we add seven other factors as control variables. However, the selected samples may have selectivity and mixed deviation, leading to bias in the benchmark regression results. The propensity score matching (PSM) method can address these problems (40), so we used the PSM on all control variables to reduce the possible differences between the treatment and control groups. We performed the logit regression on the control variables in this paper through the dummy variable (i.e., *Treat*_*it*_, it means whether the pharmaceutical firm is affected by the consistency evaluation policy of generic drugs.) and got the propensity score value, searched the pharmaceutical company with the closest propensity score value to serve as the control group that matches the treatment group. Then, we perform a balance test and co-support hypothesis test to observe whether there is a significant difference in the means of control variables between the experimental group and the control group after matching. If there is no significant difference, the PSM-DID method is justified. This paper uses the kernel matching method in the specific estimation, this method was proposed by Heckman et al. (41). When matching, the kernel matching method uses more information and has a lower variance than other methods (42), so it often appears in the literature (43–46). *Kernel matching* is a non-parametric matching method that constructs a “counterfactual” matching object for each experimental group individual by taking the weighted average of the propensity scores of all control group individuals. The kernel function and the selected bandwidth parameters jointly determine the weight assignment. The bandwidth parameter selected in this paper is 0.06 (47).

**Figure 4:**
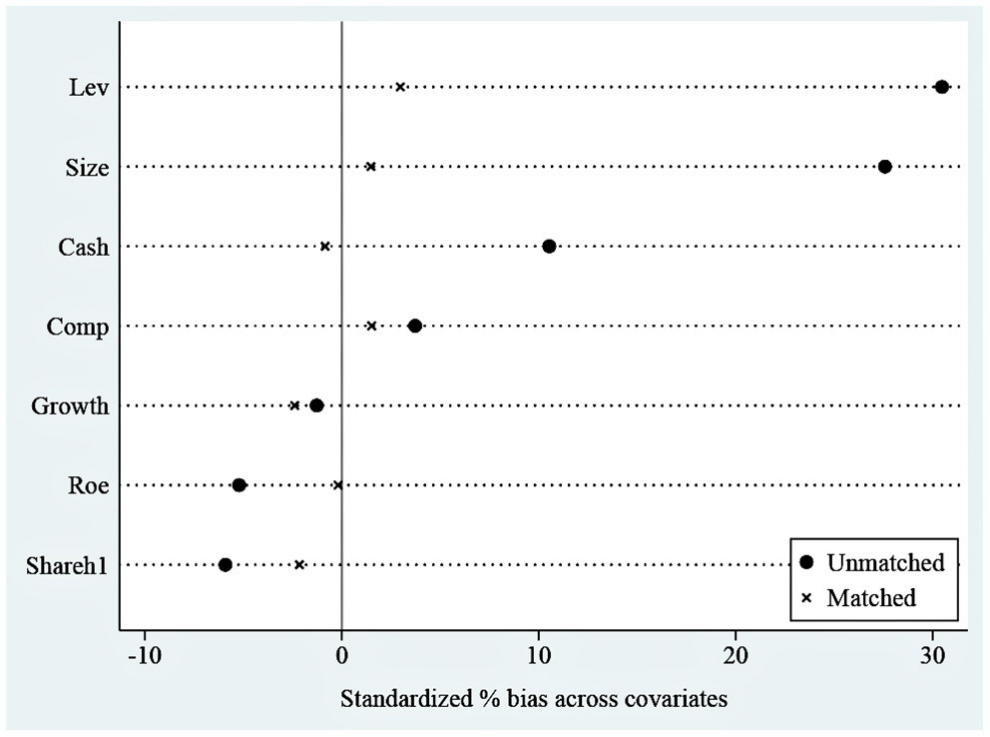
Standardized deviation before and after matching.

**Figure 5:**
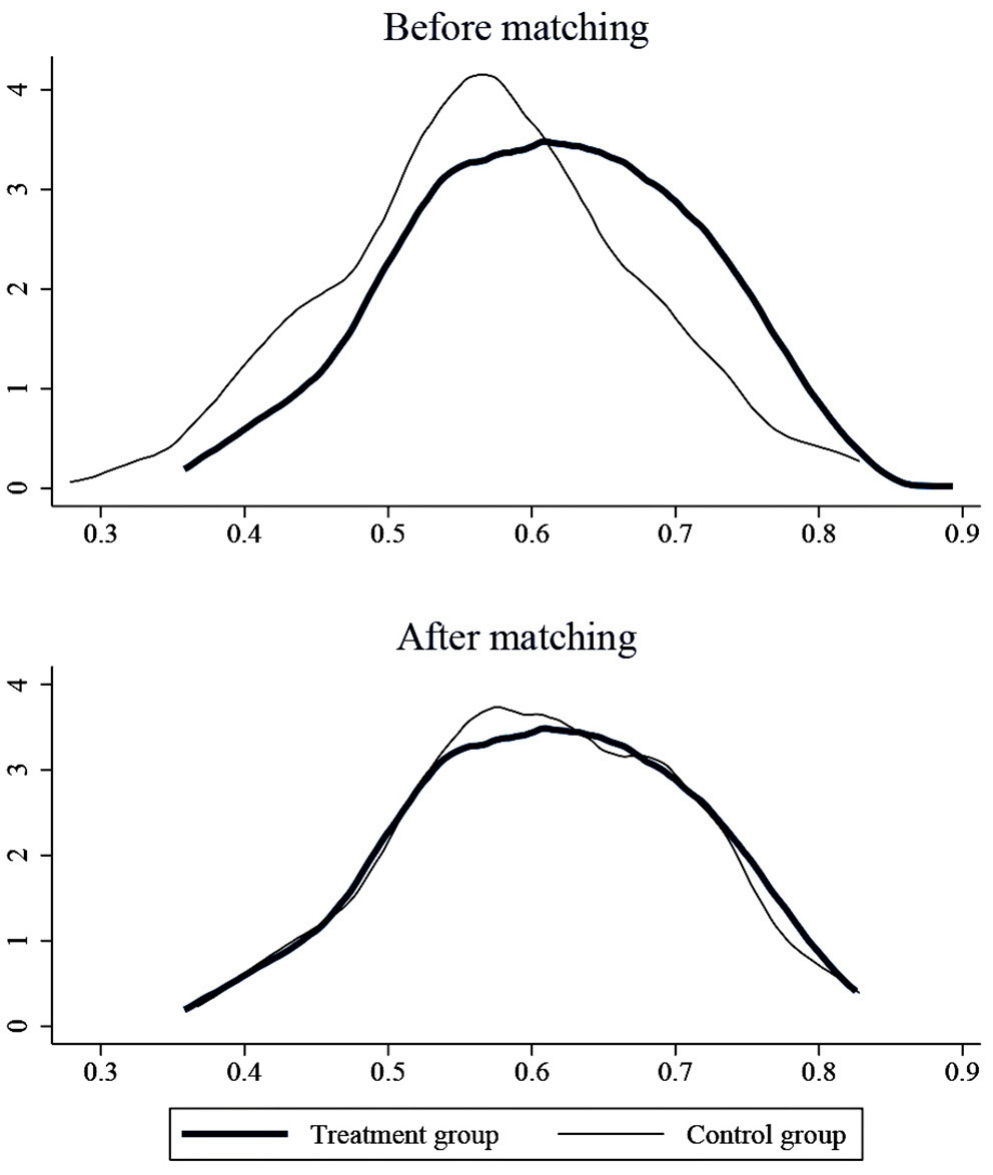
Propensity score distributions for treatment and control groups.

The results of the balance test are shown in Figure 4. As we can see, the standardized absolute difference of each variable after matching is <10%. In addition, comparing the distribution of propensity score kernel density before and after matching as shown in Figure 5, we found that after matching, the difference between the distribution of propensity scores in the treatment and control groups is significantly reduced, and the trend is the same; the data after matching is balanced, and the matching is effective. Finally, the successfully matched samples were tested in the DID model to estimate the policy's net effect.

The results of the above kernel matching were recorded in columns (1) and (2) of Table 5. We also used the K near neighbor matching method to take the *K*-value as 4 (48) and record the regression results after matching in columns (3) and (4) of Table 5. As we can see, under two matching methods, whether control variables are included or not, the policy will always have a significantly positive impact on R&D investment intensity, proving that the benchmark regression results are robust. Moreover, the core explanatory variable (*Treat*_*it*_ × *Post*_*it*_) coefficient difference between the two methods is slight.

**Table 5:** Regression results of PSM-DID.

	**Kernel matching**		**K near neighbor matching**	
	**(1)**	**(2)**	**(3)**	**(4)**
*Treat_*it*_ × Post_*it*_*	1.194^***^	1.128^***^	1.191^***^	1.133^***^
	(5.24)	(5.06)	(4.96)	(4.76)
*Constant*	4.157^***^	−3.465	4.163^***^	−2.765
	(54.76)	(−0.30)	(54.33)	(−0.26)
Observations	988	988	897	897
R-squared	0.751	0.777	0.753	0.779
Control variables	No	Yes	No	Yes
Company FE	Yes	Yes	Yes	Yes
Year FE	Yes	Yes	Yes	Yes

*Robust t-statistics in parentheses,*

*** *p < 0.01*.

### Heterogeneity Analysis

#### Operational Nature Heterogeneity

According to the operating nature of listed companies, the 111 companies studied in this paper include 32 state-owned companies, 71 private companies, and eight companies of other operational nature. Therefore, we only took the samples data of state-owned and private firms into DID model for regression and dynamic effect test. The Table 6 record the results, as shown in columns (1) and (2), the policy has no significant effect on the R&D investment intensity of state-owned enterprises. However, it can significantly promote the increase of the R&D investment intensity of private enterprises. As shown in columns (3) and (4), the impact of the policy on state-owned enterprises' R&D investment intensity had a weak positive effect in 2018, with a coefficient value of 0.811, which is significant at the 10% significance level, but this positive effect quickly disappeared again. The positive impact of the policy on the R&D investment intensity of private enterprises also began in 2018, the significance level has always been below 1%, and the coefficient of the dummy variable has been increasing. The impact of the consistency evaluation policy on the R&D investment intensity of both state-owned and private companies has a lagging effect, and the R&D investment intensity of private pharmaceutical firms is more affected by the policy of consistent evaluation for generic drugs than that of state-owned pharmaceutical enterprises. These results support hypothesis H2.

**Table 6:** Results of the operating nature heterogeneity analysis.

	**Baseline regressive results**		**Parallel trend test**	
	**State-Owned**	**Private**	**State-Owned**	**Private**
*Treat_*it*_ × Post_*it*_*	0.273	1.530^***^		
	(0.82)	(4.49)		
*Treat_, *i*_ × Post,_2013_*			0.108	−0.278
			(0.48)	(−0.70)
*Treat_, *i*_ × Post,_2014_*			0.157	−0.461
			(0.65)	(−0.86)
*Treat_, *i*_ × Post,_2015_*			−0.112	0.210
			(−0.26)	(0.54)
*Treat_, *i*_ × Post,_2016_*			−0.554	−0.033
			(−1.20)	(−0.06)
*Treat_, *i*_ × Post,_2017_*			0.385	0.565
			(0.80)	(1.09)
*Treat_, *i*_ × Post,_2018_*			0.811^*^	1.844^***^
			(2.05)	(3.83)
*Treat_, *i*_ × Post,_2019_*			0.582	2.172^***^
			(1.34)	(3.82)
*Treat_, *i*_ × Post,_2020_*			0.374	2.420^***^
			(0.73)	(4.00)
*Constant*	0.955	−4.363	1.122	−7.251
	(0.09)	(−0.23)	(0.10)	(−0.40)
Observations	288	639	288	639
R-squared	0.786	0.755	0.795	0.766
Control variables	Yes	Yes	Yes	Yes
Company FE	Yes	Yes	Yes	Yes
Year FE	Yes	Yes	Yes	Yes

*Robust t-statistics in parentheses,*

*** *p < 0.01,*

* *p < 0.1*.

#### Regional Distribution Heterogeneity

Due to the large regional heterogeneity in China, further analysis of different regional subsamples is necessary (49–51). The listed pharmaceutical companies in the sample can be divided into three groups by region: eastern, central, and western. In the sample, 66 pharmaceutical companies belong to the eastern region, 29 to the central region, and 16 to the western region. We put the relevant data of eastern, central, and western pharmaceutical companies into the DID model for benchmark regression and dynamic effect test. Columns (1-3) of Table 7 show that the generic drug consistency evaluation policy can significantly increase the R&D investment intensity of pharmaceutical companies in the east. In contrast, the policy has no significant impact on the R&D investment intensity of pharmaceutical companies in the central and western regions. As shown in columns (4-6), the positive impact of the policy on R&D investment intensity of pharmaceutical companies in the eastern region began in 2018, and the coefficient first increased and then decreased, all of which were significant at the 1% significance level; The policy had a significant positive impact on the investment intensity of drug companies in central region in 2018, and the coefficient value is 1.020, which is significant at 5% significance. However, after 2019, the impact is not significant; The policy has not shown a significant impact on the R&D investment intensity of pharmaceutical companies the western regions for the time being. In summary, the effect of the generic drug consistency evaluation policy on the R&D investment intensity of pharmaceutical companies is as follows: eastern > central and western, and the positive impact of this policy on pharmaceutical companies in the eastern will continue. Hypothesis H3 is supported.

**Table 7:** Results of the regional distribution heterogeneity analysis.

	**Baseline regressive results**			**Parallel trend test**		
	**Eastern**	**Central**	**Western**	**Eastern**	**Central**	**Western**
*Treat_*it*_ × Post_*it*_*	1.514^***^	0.304	0.336			
	(4.93)	(1.30)	(0.50)			
*Treat_, *i*_ × Post,_2013_*				−0.363	0.414	−0.277
				(−1.22)	(1.83)	(−0.30)
*Treat_, *i*_ × Post,_2014_*				−0.312	0.055	0.524
				(−0.69)	(0.07)	(0.50)
*Treat_, *i*_ × Post,_2015_*				−0.206	0.667	0.967
				(−0.43)	(1.47)	(0.74)
*Treat_, *i*_ × Post,_2016_*				0.015	0.119	1.085
				(0.03)	(0.20)	(0.45)
*Treat_, *i*_ × Post,_2017_*				0.541	0.496	0.855
				(0.83)	(1.12)	(0.65)
*Treat_, *i*_ × Post,_2018_*				1.735^***^	1.020^**^	−0.029
				(2.96)	(2.62)	(−0.04)
*Treat_, *i*_ × Post,_2019_*				2.154^***^	0.599	0.283
				(6.32)	(1.05)	(0.40)
*Treat_, *i*_ × Post,_2020_*				2.006^***^	0.753	0.967
				(4.28)	(1.82)	(0.70)
*Constant*	−10.662	1.425	14.253^*^	−9.417	−0.341	16.582^***^
	(−0.61)	(0.13)	(2.22)	(−0.52)	(−0.03)	(5.12)
Observations	594	261	144	594	261	144
R-squared	0.778	0.779	0.868	0.786	0.783	0.871
Control variables	Yes	Yes	Yes	Yes	Yes	Yes
Company FE	Yes	Yes	Yes	Yes	Yes	Yes
Year FE	Yes	Yes	Yes	Yes	Yes	Yes

*Robust t-statistics in parentheses,*

*** *p < 0.01,*

** *p < 0.05,*

* *p < 0.1*.

*According to the regional division standard of “China High-tech Industry Statistical Yearbook” before 2012: The eastern region includes: Beijing, Tianjin, Hebei, Liaoning, Shandong, Shanghai, Jiangsu, Zhejiang, Fujian, Guangdong, Guangxi, Hainan; The central region includes: Shanxi, Inner Mongolia, Jilin, Heilongjiang, Anhui, Jiangxi, Henan, Hubei, Hunan; The western region includes: Chongqing, Sichuan, Guizhou, Yunnan, Shaanxi, Gansu, Qinghai, Ningxia, Tibet, and Xinjiang*.

The impact of generic drug consistency evaluation policies on pharmaceutical companies' R&D investment presents regional heterogeneity. The reason is that most Chinese pharmaceutical companies are concentrated in the eastern region. The eastern region has three major technology R&D centers in the Yangtze River Delta (Shanghai, Jiangsu, Zhejiang) and the Pearl River Delta (Shenzhen, Guangzhou), and the spatial focus of domestic biomedical R&D investment has shifted to the eastern coastal area (52). The eastern region is mainly distributed along the coast, with flat terrain, convenient transportation, and sound economic development. It has gathered many leading pharmaceutical companies in China, with good R&D, talent and financing conditions (53), and prominent regional advantages. The pharmaceutical companies in the eastern region have more cooperation and interaction with large international pharmaceutical companies, and they are also more sensitive to changes in industry policies. China's central and western regions started late in opening to the outside world, the natural environment is poor, and the economic development is relatively slow. Especially in the western region, the infrastructure construction is relatively backward, and the technology, talents, and financing are relatively insufficient (54). The development of the pharmaceutical firms in the western is relatively backward, and there may be more obstacles for pharmaceutical companies in the western region to carry out the consistency evaluation work.

#### Profitability Heterogeneity

We divide listed pharmaceutical companies in the sample into groups according to different profitability. Kim (55) believed that ROE & ROI is the most comprehensive indicator to measure a firm's profitability. In this paper, we used return on equity (*Roe*) to represent the firm's profitability, and we used the mean value of *Roe* in the sample as the basis for grouping companies with different profitability. Because the policy started in 2016, we mainly observed the *Roe* of firms from 2016 to 2020, the *Roe* value of the firm was greater than the mean value of 8.772% for more than 4 years, and there was no negative value in every year. It was divided into the sample group with high profitability, and finally, we got 46 companies. The remaining 65 companies were served as the sample group with low profitability. We put the relevant data of the two sample groups into the DID model for benchmark regression and parallel trend test, respectively, and the results are shown in Table 8. From the benchmark regression in columns (1) and (2), it can be seen from the average effect that regardless of whether the profitability of pharmaceutical companies is high or low, their R&D investment intensity has increased significantly under the background of the generic drug consistency evaluation policy. However, the policy affects the R&D investment of firms with high profitability more than those with low profitability. As shown in columns (3) and (4), the positive impact of generic consistency evaluation policy on R&D investment of pharmaceutical firms with different profitability began in 2018. The positive impact of policies on the R&D investment intensity of high-profitability pharmaceutical companies is increasing. In 2018 and 2019, the coefficient is significant at 5%, and the coefficient is significant at 1% in 2020. The positive impact of the policy on the R&D investment intensity of low-profitability pharmaceutical companies increases first and then decreases. In 2018 and 2020, the coefficient is significant at 5%, and the coefficient is significant at 1% in 2019. Moreover, the coefficients of low-profitability are all lower than those of high-profitability. In summary, the consistency evaluation policy of generic drugs has a greater impact on the R&D investment intensity of firms with high profitability. The research hypothesis H4 is proved.

**Table 8:** Results of profitability heterogeneity analysis.

	**Baseline regressive results**		**Parallel trend test**	
	**High-Profitability**	**Low-Profitability**	**High-Profitability**	**Low-Profitability**
*Treat_*it*_ × Post_*it*_*	1.324^***^	0.945^***^		
	(3.34)	(3.54)		
*Treat_, *i*_ × Post,_2013_*			0.085	−0.426
			(0.26)	(−1.53)
*Treat_, *i*_ × Post,_2014_*			−0.404	−0.111
			(−0.50)	(−0.35)
*Treat_, *i*_ × Post,_2015_*			0.135	−0.149
			(0.18)	(−0.40)
*Treat_, *i*_ × Post,_2016_*			−0.264	−0.028
			(−0.27)	(−0.08)
*Treat_, *i*_ × Post,_2017_*			0.298	0.371
			(0.40)	(0.77)
*Treat_, *i*_ × Post,_2018_*			1.642^**^	1.102^**^
			(2.22)	(2.15)
*Treat_, *i*_ × Post,_2019_*			2.052^**^	1.304^***^
			(2.72)	(3.22)
*Treat_, *i*_ × Post,_2020_*			2.653^***^	1.159^**^
			(3.47)	(2.21)
*Constant*	−2.969	−3.714	−7.729	−3.992
	(−0.11)	(−0.47)	(−0.30)	(−0.49)
Observations	414	585	414	585
R-squared	0.816	0.719	0.828	0.723
Control variables	Yes	Yes	Yes	Yes
Company FE	Yes	Yes	Yes	Yes
Year FE	Yes	Yes	Yes	Yes

*Robust t-statistics in parentheses,*

*** *p < 0.01,*

** *p < 0.05.*

*The Roe is not included in the control variables*.

## Conclusion and Discussion

The quality and efficacy consistency evaluation policy of generic drugs is vital to ensure the high-quality development of generic medicines in China. It will also change the competition pattern of the whole pharmaceutical industry in China and have a profound impact on enterprise research and development activities. In this paper, we conduct a quasi-natural experiment on the policy and use the difference-in-differences method to analyze its effect on pharmaceutical companies' R&D investment empirically. The results show that the policy positively impacts the R&D investment intensity, and the impact is lagging by 2 years. In the early stage of the implementation of the policy, most companies were waiting and watching, so the R&D investment of most generic drug companies did not change much in the first 2 years. However, the test of parallel trend shows that the positive effect of this policy on the R&D investment intensity of firms is still increasing with time. We tested the baseline regression results by placebo test and PSM-DID test of the sample, and the test results show that the baseline regression results are robust. We also conducted heterogeneity analyses of the impact of the policy on the R&D investment of pharmaceutical companies. The R&D investment intensity of private pharmaceutical companies is more affected by the policy than that of the state-owned pharmaceutical companies, private enterprises are more dependent on the institutional environment, so they are more sensitive to the policy. The impact of the generic drug consistency evaluation policy on the R&D investment intensity of pharmaceutical enterprises in the eastern region is greater than that of the pharmaceutical firms in the central and western regions. Because different pharmaceutical companies have different products, different amounts of investment, different R&D capabilities, and different responses to the policy, the policy affects the R&D investment intensity of pharmaceutical companies of different operation nature and regions show differences. There are geographical advantages in the eastern region pharmaceutical companies of China; its climate and economic conditions are more suitable for the development of pharmaceutical companies. The pharmaceutical companies in the eastern region are relatively concentrated and have a high response to industry policies. Regardless of the profitability of enterprises, the policy will significantly affect the R&D investment of pharmaceutical firms, and this effect is positive. At the same time, the R&D investment intensity of firms with high profitability is more affected by the policy, proving that firms with high profitability have more advantages in fierce competition, and companies with high profitability can flexibly adjust their R&D investment strategies according to the policy.

On March 11, 2022, National Medical Products Administration (56) released the notice of “Catalog of Chemical Generic Drug Reference Preparations (Fiftyth Batch)” (No. 16 of 2022). That means the relevant support measures for Chinese generic drug quality and efficacy consistency evaluation policy gradually improve. The quality and efficacy consistency evaluation of generic drugs will become the path that the development of generic drug companies must go through. In addition, foreign pharmaceutical firms that have passed the consistency evaluation of generic drugs have also begun to appear in the Chinese Chemicals Catalog, proving the scope of influence of the consistency evaluation policy of generics is expanding. In other words, the storm caused by the consistent evaluation of Chinese generic drugs has also affected the development of foreign pharmaceutical companies in China. If they want to obtain some shares in pharmaceutical market of China, they should participate in consistent evaluation activities with domestic enterprises.

For the above research conclusions, we have drawn the following policy implications: ①The generic drug quality and efficacy consistency evaluation policy is an indirect innovation incentive policy, and as an external factor of the enterprise, it can significantly affect the enterprise's R&D investment intensity in the short term and force the enterprise to transform and upgrade, but its long-term impact remains to be studied. ②Related supporting regulations and policies for the quality and efficacy consistency evaluation of generic drugs still need to be improved, and policy implementation and incentives need to be increased to promote non-profit-oriented state-owned enterprises to increase investment in innovative R&D under policy incentives. ③The ultimate goal of the consistency evaluation policy is to improve the quality of generic drugs, promote the overall transformation and upgrading of the Chinese pharmaceutical industry, and improve the international competitiveness of Chinese pharmaceutical companies. Under the policy, the concentration of the pharmaceutical industry has been further improved, and the concentration advantage has appeared. However, it is also necessary to consider the regional differences in policy implementation. Moreover, the pharmaceutical firms participating in the consistency evaluation in the central and western regions need more government support to promote the development of firms to a high-quality level, such as by increasing government subsidies or helping establish a platform for cooperation and exchange of pharmaceutical companies. ④Relevant departments should publicize the generic drugs that have passed the evaluation, actively support the use of these generic drugs in clinical, and drive pharmaceutical companies to participate in the evaluation actively. In terms of centralized procurement, the restrictions on the number of bid-winning enterprises should be appropriately loosened. Then, the profitability of more companies is improved, making them more capable of R&D innovation activities.

Compared with previous studies by scholars, this paper provides empirical evidence for the consistency evaluation policy generic drugs to encourage pharmaceutical firms to increase R&D investment and conduct an in-depth analysis of the impact of the policy from the perspective of heterogeneity. However, because we use balanced panel data and the financial data disclosure of many pharmaceutical firms is not complete, the sample size we used for the research is small. Moreover, the size of the firms in the sample is relatively close, potentially affecting the conclusions. These are the limitation of this research, and the sample size needs to expand for further study in the future. Regarding heterogeneity analysis, the sample of state-owned enterprises and the central and western regions samples are relatively small, and the sample size can be increased for subsequent research. The increase in R&D investment will also drive the increase in the R&D output of pharmaceutical companies. If we find enough data, we will continue to conduct in-depth research on the policy impact on R&D output (such as the number of patent applications) and conduct in-depth research on the differences in policy impact on pharmaceutical firms according to their financial status. We will also conduct an in-depth analysis of the specific mechanism of promoting the pharmaceutical firm's R&D investment under the policy.

## Data Availability Statement

The original contributions presented in the study are included in the article/Supplementary Material, further inquiries can be directed to the corresponding author/s.

## Author Contributions

YW and YC: conceptualization and methodology. YW and JZ: data curation. JZ and JQ: formal analysis. YW: software. DZ and YC: supervision and writing–review and editing. YW, JZ, and JQ: writing–original draft preparation. All authors have read and agreed to the published version of the manuscript.

## Funding

We gratefully acknowledge the financial support from Liaoning Association for Science and Technology (Grant No. LNKX2021B03).

## Conflict of Interest

The authors declare that the research was conducted in the absence of any commercial or financial relationships that could be construed as a potential conflict of interest.

## Publisher's Note

All claims expressed in this article are solely those of the authors and do not necessarily represent those of their affiliated organizations, or those of the publisher, the editors and the reviewers. Any product that may be evaluated in this article, or claim that may be made by its manufacturer, is not guaranteed or endorsed by the publisher.

## References

[1] The Office of the State Council of China. Notice of the State Council on the Issuance of the National Drug Safety Twelfth Five-Year Plan [2012] No. 5. The Office of the State Council of China (2012). Available online at: http://www.gov.cn/zwgk/2012-02/13/content_2065197.htm (accessed October 16, 2021).

[2] The Office of the State Council of China. Opinions of the General Office of the State Council on the Consistency Evaluation of the Quality and Efficacy of Generic Drugs [2016] No. 8. The Office of the State Council of China (2016). Available online at: http://www.gov.cn/zhengce/content/2016-03/05/content_5049364.htm (accessed November 12, 2021).

[3] Huang B Barber SL Xu M Cheng S. Make up a missed lesson-New policy to ensure the interchangeability of generic drugs in China. Pharmacol Res Perspect. (2017) 5:e00318. 10.1002/prp2.318 28603636 PMC5464346

[4] Hu L Yu Z. Generics industry impact study of consistency evaluation. Chin J Pharm. (2016) 47:1097–101. 10.16522/j.cnki.cjph.2016.08.030

[5] He D. Analysis of policy impact of consistency evaluation for generic drugs on the pharmaceutical industry. J Tradit Chin Med. (2017) 25:12–14. 10.16690/j.cnki.1007-9203.2017.14.006

[6] Wang J Yang Y Xu L Shen Y Wen X Mao L. Impact of '4+7' volume-based drug procurement on the use of policy-related original and generic drugs: a natural experimental study in China. BMJ Open. (2022) 12:e054346. 10.1136/bmjopen-2021-054346 35288385 PMC8921850

[7] The National Food Drug Administration of China. Notice of the National Food and Drug Administration on Quality Consistency Evaluation of Generic Drugs [2013] No. 34. The National Food and Drug Administration of China (2013). Available online at: https://www.nmpa.gov.cn/xxgk/fgwj/gzwj/gzwjyp/20130216144301191.html (accessed October 17, 2021).

[8] The China's National Health Commission & State Administration of Traditional Chinese Medicine. Notice on Issuing the National Essential Medicines List (2018 Edition) [2018] No. 31. The China's National Health Commission & State Administration of Traditional Chinese Medicine (2018). Available online at: http://www.gov.cn/zhengce/zhengceku/2018-12/31/content_5435470.htm (accessed April 13, 2021).

[9] National Medical Products Administration. Announcement on Matters Related to the Quality and Efficacy Consistency Evaluation of Generic Drugs. [2018] No. 102. National Medical Products Administration (2018). Available online at: https://www.nmpa.gov.cn/xxgk/ggtg/qtggtg/20181228161301646.html (accessed April 13, 2021).

[10] National Medical Products Administration. Announcement on Carrying out the Quality and Efficacy Consistency Evaluation of Generic Drugs for Chemical Injections. [2020] No. 62. National Medical Products Administration (2020). Available online at: https://www.nmpa.gov.cn/xxgk/ggtg/qtggtg/20200514162201667.html [Accessed October 23, 2021].

[11] Yang Q Liu L Zhou B. Analysis on the evolution and evaluation methods of the consistency evaluation. CJPH. (2019) 50:338–44. 10.16522/j.cnki.cjph.2019.03.015

[12] Cao S Jiang J. The influence of national generic drug consistency evaluation policy on drug production enterprises and countermeasures. Manage Technol SME. (2017) 2017:135–6.

[13] Liu L Yu B. How to keep advantages after generic drug quality equivalence assessment. China J Pharm Econ. (2018) 13:98–102. 10.12010/j.issn.1673-5846.2018.06.027

[14] Hu Y Zong X Yu M Li Y. Analysis of the current environmental situation of China's generic drug consistency evaluation policy. Chin J Drug Evaluat. (2020) 37:321–6.

[15] The Office of the State Council of China. The Notice on Printing and Distributing the Pilot Program for the Centralized Purchasing and Use of Drugs Organized by the State. [2019] No. 2. The Office of the State Council of China (2019). Available online at: http://www.gov.cn/zhengce/content/2019-01/17/content_5358604.htm (accessed April 16, 2021).

[16] National Health Insurance Administration. The Implementation Opinions on Expanding the Regional Scope of the Pilot Program of Centralized Drug Purchasing and Use of Drugs Organized by the State. [2019] No. 56. National Health Insurance Administration (2019). Available online at: http://www.gov.cn/zhengce/zhengceku/2019-09/30/content_5456439.htm (accessed April 16, 2021).

[17] Alam A Uddin M Yazdifar H. Financing behaviour of R&D investment in the emerging markets: the role of alliance and financial system. R&D Manage. (2017) 49:21–32. 10.1111/radm.12303

[18] Wang J. Innovation and government intervention: a comparison of Singapore and Hong Kong. Res Policy. (2018) 47:399–412. 10.1016/j.respol.2017.12.00832138748

[19] Xu J Wang X Liu F. Government subsidies, R&D investment and innovation performance: analysis from pharmaceutical sector in China. Technol Anal Strat Manage. (2020) 33:535–53. 10.1080/09537325.2020.1830055

[20] Zhou Z Wang Y Lu M Zhu J Wang S. Government intervention, financial support, and comprehensive efficiency of enterprise independent innovation: empirical analysis based on the data of chinese strategic emerging industries. Math Probl Eng. (2020) 2020:8723062. 10.1155/2020/8723062

[21] Wang L. Measurement and empirical analysis of enterprise innovation-driven growth. Stat Res. (2015) 32:62–8. 10.19343/j.cnki.11-1302/c.2015.08.008

[22] Shi S Ke J Wu X. Performance analysis of innovation development of state-owned pharmaceutical enterprises in China. Chin J New Drugs. (2020) 29:1081–6.

[23] Wang S Su Y Chen Y. Comparative study of technological innovation efficiency of pharmaceutical manufacturing in eastern, central and western regions of China. Her Med. (2016) 35:1276–80. 10.3870/j.issn.1004-0781.2016.11.031

[24] Wang W Yu B Yan X Yao X Liu Y. Estimation of innovation's green performance: a range-adjusted measure approach to assess the unified efficiency of China's manufacturing industry. J Clean Prod. (2017) 149:919–24. 10.1016/j.jclepro.2017.02.174

[25] Chen B Lee K. Cash flow, R&D investment and profitability: evidence from Chinese high-tech and other industrial firms. Korea Int Trade Res Inst. (2018) 14:51–65. 10.16980/jitc.14.2.201804.51

[26] Dalvadi YM Mansuri SI. A study on impact of profitability and R&D intensity on R&D expenditure in selected pharmaceutical companies. Al-Barkaat J Fin Manage. (2018) 10:25–33. 10.5958/2229-4503.2018.00003.6

[27] Duan T Zhang X Hu Y. A study of the threshold effect of R&D intensity and the performance of China' listed pharmaceutical manufacturers. Manage Rev. (2020) 32:142–52. 10.14120/j.cnki.cn11-5057/f.2020.09.0127381264

[28] Si Y. Analysis of Impact of Generic Consistence Evaluation Policy on Pharmaceutical Companies——Based on the Perspective of the Stock Price and R&D Investment. (Master's Thesis), Southwestern University of Finance and Economics, Chengdu (China) (2019).

[29] Majumdar SK. Scalability versus flexibility: firm size and R&D in Indian industry. J Technol Transf. (2011) 36:101–16. 10.1007/s10961-009-9147-x

[30] Wen H Xia K. Venture capital, ownership concentration and enterprise R&D investment. Proc Comp Sci. (2016) 91:519–25. 10.1016/j.procs.2016.07.133

[31] Jung S Kwak G. Firm characteristics, uncertainty and research and development (R&D) investment: the role of size and innovation capacity. Sustainability. (2018) 10:1668. 10.3390/su1005166835393231

[32] Yang J Wang L Sun Z Zhu F Guo Y Shen Y. Impact of monetary policy uncertainty on R&D investment smoothing behavior of pharmaceutical manufacturing enterprises: empirical research based on a threshold regression model. Int J Environ Res Public Health. (2021) 18:11560. 10.3390/ijerph182111560 34770073 PMC8583370

[33] Rojas C. Market power and the lerner index: a classroom experiment. J Indust Organ Educ. (2011) 5:1–19. 10.2202/1935-5041.1033

[34] Bertrand M Duflo E Mullainathan S. How much should we trust differences-in-differences estimates? SSRN Electro J. (2001) 119:249–75. 10.2139/ssrn.288970

[35] Liu Q Qiu LD. Intermediate input imports and innovations: evidence from Chinese firms' patent filings. J Int Econ. (2016) 103:166–83. 10.1016/j.jinteco.2016.09.009

[36] Lyu Y Lu Y Wu S Wang Y. The effect of the belt and road initiative on firms' OFDI: evidence from China's greenfield investment. Econ Res J. (2019) 54:187–202. Available online at: https://kns.cnki.net/kcms/detail/detail.aspx?FileName=JJYJ201909013&DbName=CJFQ20

[37] La Ferrara E Chong A Duryea S. Soap operas and fertility: evidence from Brazil. Appl Econ. (2012) 4:1–31. 10.1257/app.4.4.1

[38] Liu Q Lu Y. Firm investment and exporting: evidence from China's value-added tax reform. J Int Econ. (2015) 97:392–403. 10.1016/j.jinteco.2015.07.003

[39] Cai X Lu Y Wu M Yu L. Does environmental regulation drive away inbound foreign direct investment? Evidence from a quasi-natural experiment in China. J Dev Econ. (2016) 123:73–85. 10.1016/j.jdeveco.2016.08.003

[40] Rosenbaum P Rubin D. The central role of the propensity score in observational studies for causal effects. Biometrika. (1983) 70:41–55. 10.1093/biomet/70.1.41

[41] Heckman J Ichimura H Todd P. Matching as an econometric evaluation estimator: evidence from evaluating a job training programme. Rev Econ Stud. (1997) 64:605–54. 10.2307/2971733

[42] Cheng L Liu H Zhang Y Shen K Zeng Y. The impact of health insurance on health outcomes and spending of the elderly: evidence from China's new cooperative medical scheme. Health Econ. (2015) 24:672–91. 10.1002/hec.3053 24777657 PMC4790431

[43] Chen Y Xu C Yi M. Does the belt and road initiative reduce the R&D investment of OFDI enterprises? Evidence from China's A-share listed companies. Sustainability. (2019) 11:1321. 10.3390/su11051321

[44] Wang H Chen Z Wu X Nie X. Can a carbon trading system promote the transformation of a low-carbon economy under the framework of the porter hypothesis?—Empirical analysis based on the PSM-DID method. Energy Policy. (2019) 129:930–8. 10.1016/j.enpol.2019.03.007

[45] Tang K Liu Y Zhou D Qiu Y. Urban carbon emission intensity under emission trading system in a developing economy: evidence from 273 Chinese cities. Environ Sci Pollut Res. (2021) 28:5168–79. 10.1007/s11356-020-10785-1 32959321

[46] Qin J Cao J. Carbon emission reduction effects of green credit policies: empirical evidence from China. Front Environ Sci. (2022) 10:798072. 10.3389/fenvs.2022.798072

[47] Su D Chen YC Gao HX Li HM Chang JJ Jiang D. Effect of integrated urban and rural residents medical insurance on the utilisation of medical services by residents in China: a propensity score matching with difference-in-differences regression approach. BMJ Open. (2019) 9:e026408. 10.1136/bmjopen-2018-026408 30782944 PMC6377539

[48] Zang J Wan L Li Z Wang C Wang S. Does emission trading scheme have spillover effect on industrial structure upgrading? Evidence from the EU based on a PSM-DID approach. Environ Sci Pollut Res Int. (2020) 27:12345–57. 10.1007/s11356-020-07818-0 31993897

[49] Nie X Wu J Zhang W Zhang J Wang W Wang Y. Can environmental regulation promote urban innovation in the underdeveloped coastal regions of western China? Mar Policy. (2021) 133:104709. 10.1016/j.marpol.2021.104709

[50] Nie X Wu J Chen Z Zhang A Wang H. Can environmental regulation stimulate the regional porter effect? Double test from quasi-experiment and dynamic panel data models. J Clean Prod. (2021) 314:128027. 10.1016/j.jclepro.2021.128027

[51] Nie X Wu J Wang H Li L Huang C Li W. Booster or stumbling block? The role of environmental regulation in the coupling path of regional innovation under the porter hypothesis. Sustainability. (2022) 14:2876. 10.3390/su14052876

[52] Gu J. Determinants of biopharmaceutical R&D expenditures in China: the impact of spatiotemporal context. Scientometrics. (2021) 126:6659–80. 10.1007/s11192-021-04058-y 34188331 PMC8221558

[53] Ma J Seong Y Lee M. Evaluation of resource-based provincial innovation environment index system based on open innovation theory. Innov Stud. (2021) 16:77–96. 10.46251/INNOS.2021.11.16.4.77

[54] Xie R. Under the background of “one belt and one road,” China's central and western regions open to the outside world. China Collect Econ. (2019) 36:38–. Available online at: https://kns.cnki.net/kcms/detail/detail.aspx?FileName=ZJTG201936015&DbName=CJFQ2019

[55] Kim HS. A study of financial performance using dupont analysis in food distribution market. Culin Sci Hosp Res. (2016) 22:52–60. 10.20878/cshr.2016.22.6.005

[56] National Medical Products Administration. Announcement of the National Medical Products Administration on Issuing the Catalogue of the Generic Drug Reference Preparations (Fiftyth Batch). National Medical Products Administration (2022). Available online at: https://www.nmpa.gov.cn/xxgk/ggtg/qtggtg/20220111084200136.html (accessed March 12, 2022).

