# Supplementary material for: The Impact of the Consistency Evaluation Policy of Generic Drugs on R&D Investment Intensity of Pharmaceutical Companies—An Empirical Study Based on the Difference-in-Differences Model
Source: Front Public Health. 2022 Jun 9;10:902025. doi: 10.3389/fpubh.2022.902025 (PMC9218526; doi:10.3389/fpubh.2022.902025)
Supplement: Supplementary file 1 [file Table_1.DOCX]

Supplementary Material

# Supplementary Table

**Table a│ Regulations and policies related to the consistency evaluation of generic drug**

| **Date** | **Department** | **policy** | **Key points** |
| --- | --- | --- | --- |
| January 20, 2012 | Office of the State Council | The 12th Five-Year Plan for National Drug Safety | The quality of generic drugs ought to be improved, and the consistency evaluation of generic drugs ought to carry out vigorously. |
| August 9, 2015 | Office of the State Council | Opinions on reforming the review and approval system for drugs and medical devices | The definition of generic drugs changed from imitating drugs with national standards to those with the same quality and efficacy as the original drugs. |
| March 5, 2016 | Office of the State Council | Opinions on Carrying out the Quality and Efficacy Consistency Evaluation of Generic Drugs | It clearly stated that generic drugs approved for marketing before the implementing of the new registration classification for chemical drugs, including domestic generic drugs and imported generic drugs, shall be subject to consistency evaluation. |
| March 9, 2016 | CFDA | Report on the reform of the registration and classification of chemical drugs (No. 51 of 2016) | The new registration classification of chemical drugs started. Generic drugs are devised as "drugs that imitate overseas marketed but un-marketed original drugs in China" and "drugs that imitate Chinese marketed original drugs." |
| March 18, 2016 | CFDA | Notice on the release of three technical guidelines for the selection and determination of reference preparations for ordinary oral solid preparations | The purpose of these documents is to provide technical guidance for the selection and determination of generic drug reference preparations and human bioequivalence studies of generic drugs. |
| May 18, 2016 | CFDA | Notice on Issuing Guidelines for Human Bioequivalence Test Exemptions | This document publishes exemption requirements for bioequivalence testing of BCS Class 1 and 3 drugs and recommends the methods for classifying drug substances and determining drug product dissolution properties. |
| May 18, 2016 | CFDA | Announcement on the issuance of procedures for registration and recommendation of reference preparations for quality and efficacy consistency evaluation of generic drugs | It published the registration and recommendation procedures of the reference preparation and the requirements for the submission of reference preparation. |
| May 25, 2016 | CFDA | Announcement of a working procedure for quality and efficacy consistency evaluation of generic drugs | It published work procedures for consistency evaluation data declaration, data acceptance, clinical trial data verification, drug review inspection, technical review, and other procedures. |
| May 25, 2016 | CFDA | Announcement on the implementation of the ' Views of the Office of the State Council on the consistency evaluation of the quality and efficacy of generic drugs ' | It announced a catalog of 289 varieties of generic drugs that must be evaluated for consistency by the end of 2018. |
| August 25, 2017 | CFDA | Announcement on matters related to the consistency evaluation of the quality and efficacy of generic drugs | It provides advice and guidance for firms to select reference preparations and apply for exemptions from human bioequivalence studies. |
| December 29, 2017 | CDE | Chinese List of Chemical Drugs | The list records the medicines registered under the new registration classification and contains information about the generics that have passed the consistency evaluation. This list can also be called "China's Orange Book". |
| April 3, 2018 | Office of the State Council | Opinions on Reforming and Perfecting the Supply Guarantee and Use Policy of Generic Drugs | It is recommended to formulate a drug catalog that encourages generic drugs, strengthens the technical research of generic drugs, further improves the quality and clinical efficacy of generic drugs, improves relevant policies, and supports the development of generic drugs. |
| March 28, 2019 | NMPA | Announcement on Issuing the Selection and Determination Procedures for Reference Preparations of Chemical Generic Drugs | The selection procedures for reference preparations are unified to avoid new problems in the selection of reference preparations and ensure that the quality of the generic drugs is consistent with the original drugs from the initial stage. |
| May 14, 2020 | NMPA | Announcement on Carrying out the Quality and Efficacy Consistency Evaluation of Generic Drugs for Chemical Injections | The consistency evaluation of generic injection drugs officially started. |

**Note:** CFDA: Chinese Food and Drug Administration, the use of CFDA since 2013;

CDE: The Center for Drug Evaluation of China;

NMPA: National Medical Products Administration, NMPA has been used since September 2018. Considering the particularity of drug regulation, the Chinese government has set up this separate drug supervision bureau.

BCS：Biopharmaceutical Classification System.
